# Age impact on the eferent system actitivies in cochlear mechanical properties in normal hearing individuals

**DOI:** 10.1016/S1808-8694(15)30648-0

**Published:** 2015-10-19

**Authors:** Jerusa Roberta Massola de Oliveira, João Candido Fernandes, Orozimbo Alves Costa Filho

**Affiliations:** 1 Doctoral degree. Speech therapist of the Hospital de Reabilitação de Anomalias Craniofaciais, Universidade de Sao Paulo; 2 Full professor, Department of Mechanical Engineering, Universidade Estadual Paulista; 3 Full professor, Department of Speech Therapy, Universidade de Sao Paulo - Campus Bauru. Vice-Coordinator of the Centro de Pesquisas Audiológicas, Universidade de Sao Paulo

**Keywords:** hearing, aging, organ of corti

## Abstract

The medial olivocochlear tract has efferent control over the outer hair cells, regulating the slow contractions and damping the fast ones. Using ipsilateral, contralateral or bilateral otoacoustic emissions amplitude studies, it is possible to estimate the conditions of this tract, since the effect resulting from the reduction/suppression of these emissions indicate the tract's functioning. Aging implies an activity reduction in the central auditory system, because of the degeneration of the structures involved in hearing skills.

**Aim:**

our goal was to investigate the effects of age on the activities of this tract on the cochlea, through the analysis of the emissions' amplitude with contralateral acoustic stimulation.

**Materials and methods:**

Our series was made up of 75 individuals grouped according to age. The methodology was conventional, with a linear click and a white noise.

**Case study:**

the analysis considered the response from both ears and the comparison between the groups.

**Results:**

the results show a statistically significant difference between the emissions' response with and without contralateral acoustic stimulation in the individuals between 20 and 39 years of age. The emissions reduction/suppression effect reduced with age (fourth decade).

**Conclusion:**

aging impairs the tract effectiveness.

## INTRODUCTION

The afferent and efferent auditory systems monitor the outer hair cell electromotility. The afferent innervation transmits information to the brain about the strain, length and stiffness of the outer hair cells. The efferent innervation, consisting of the medial olivocochlear efferent tract, regulates the slow contractions of the outer hair cells and attenuates the rapid contractions by increasing the impedance of the system, which dampens and reduces the amplitude of otoacoustic emissions.

The medial olivocochlear efferent tract originates from the area around the superior olivary complex, and is composed of thicker myelinated fibers that course mostly (72% to 74%) to the contralateral cochlea, ending on the outer hair cells; the remaining fibers (26% to 28%) course to the ipsilateral cochlea.^1^ Otoacoustic emissions with an acoustic stimulus may be used clinically to objectively study the integrity of this tract.^2^

Suppression of otoacoustic emissions or the efferent olivocochlear reflex may be characterized by amplitude suppression or altered latency and phase change of evoked otoacoustic emissions when a contralateral, ipsilateral or bilateral acoustic stimulus to the tested ear is presented as recording is done.^3^

The medial olivocochlear efferent tract is affected as the auditory system undergoes peripheral and central anatomical and structural changes during the aging process; such changes take place in the afferent and efferent pathways. Central alterations extend from the auditory nerve to the central auditory cortex, and include neuronal demyelination.^4^,^5^

Auditory pathway structures begin to degenerate at age 40 years; the myelin sheath of its neurons start to deteriorate, which reduces the functional effectiveness of these pathways.^4^

An international study of elderly subjects showed that the suppression effect decreases with age, suggesting that loss of function of the tract may be related with aging.^6^ Parthasarathy studied adult and elderly subjects and also found a decreased suppression of otoacoustic emissions as age progressed.^7^

The purpose of this study was to investigate the influence of age on the activity of the medial olivocochlear efferent tract and the mechanical properties of the cochlea, by analyzing the amplitude of otoacoustic emissions with contralateral stimulation in normal hearing subjects.

## MATERIAL AND METHOD

The study was done according to the Research Ethics Committee rules for research on human being of the graduate course of our institution; the approval document was numbered 298/2003-UEP-CEP.

Inclusion criteria included: normal results in the audiological evaluation (pure tone audiometry, logoaudiometry and acoustic immittance testing), and transient evoked otoacoustic emissions without contralateral acoustic stimulation. The series consisted of 75 adult subjects of both sexes divided into: group 1 (20 – 30 years), group 2 (30 – 40 years), group 3 (40 – 50 years), group 4 (50 – 60 years), and group 5 (over 60 years).

Investigation procedures for transient evoked otoacoustic emissions were: the presence of transient evoked otoacoustic emissions, and recording of transient evoked otoacoustic emissions without and with contralateral acoustic stimulation, measured on an Otodynamics two-channel analyzer (ILO 292 DP ECHO Research OAE System) with two acoustic probes (A and B).

First, the presence of transient evoked otoacoustic emissions with conventional mode recording was verified. The A probe emitted the evoking acoustic stimulus (non-linear click at 80 dBSPL - ±1) for transient evoked otoacoustic emissions. Recording of transient evoked otoacoustic emissions was done first in the right ear.

Then, transient evoked otoacoustic emissions without and with contralateral acoustic stimulation were recorded using the conventional mode. The evoking acoustic stimulus originated in the A channel (linear click at 60 dBSPL - ±1) without and with contralateral acoustic stimulation. With contralateral acoustic stimulation, white noise was presented in the B channel (at 60 dBSPL - ±1).

Transient evoked otoacoustic emissions and transient evoked otoacoustic emissions without and with contralateral acoustic stimulation parameter verification and recording procedures were: reproducibility (70%) and analysis time (2.5 ms to 20 ms). The defining criterion for the presence of transient evoked otoacoustic emissions was amplitude of otoacoustic emissions equal to or higher than 3 dBSPL above noise in at least three consecutive frequencies.^8^

Reduction/suppression was defined as the amplitude difference (dBSPL) of otoacoustic emission responses without and with contralateral acoustic stimulation. Reduction was defined as present when the difference was positive, with an amplitude response reduction of transient evoked otoacoustic emissions with contralateral acoustic stimulation. Suppression was defined as present when transient evoked otoacoustic emission responses were extinguished. Reduction/suppression of transient evoked otoacoustic emissions was absent when the difference was zero or absent.

The analysis of variance for repeated measures^9^ was applied in the inferential analysis to study the association among transient evoked otoacoustic emission results and the correlation among the factors: ear, group, and noise. Bonferroni's correction was applied for post hoc comparisons.

## RESULTS

There were 55 male and 20 female normal-hearing subjects in the analysis.

Table 1 shows the estimated mean confidence intervals and the standard error, and the confidence interval showing the upper and lower limit for all of the groups studied without and with contralateral acoustic stimulation.

**Table 1:** Estimated mean confidence intervals, standard error and confidence interval.

Group	Ear	Noise	Estimated mean	Standard error	Confidence interval* (95%)	
					Lower limit	Upper limit
20| –30	LE	no noise	7,12	0,77	5,57	8,66
		with noise	6,68	0,77	5,13	8,22
	RE	no noise	8,23	0,98	6,25	10,20
		with noise	7,31	0,96	5,38	9,24
30| –40	LE	no noise	5,60	0,69	4,22	6,98
		with noise	4,51	0,69	3,13	5,89
	RE	no noise	6,45	0,88	4,69	8,22
		with noise	5,82	0,86	4,09	7,55
40| –50	LE	no noise	3,89	0,85	2,19	5,59
		with noise	3,47	0,84	1,78	5,16
	RE	no noise	5,06	1,11	2,84	7,28
		with noise	4,53	1,09	2,35	6,70
50| –60	LE	no noise	3,82	0,79	2,24	5,39
		with noise	3,64	0,79	2,06	5,22
	RE	no noise	4,90	0,97	2,97	6,83
		with noise	4,34	0,94	2,45	6,22
60 or more	LE	no noise	3,26	0,76	1,74	4,77
		with noise	3,23	0,76	1,72	4,74
	RE	no noise	2,84	0,99	0,86	4,82
		with noise	2,78	0,97	0,85	4,72

Table 2 shows the results of post hoc comparisons, where statistically significant differences were found only in groups 1 and 5 for the results without contralateral acoustic stimulation. Statistically significant differences were also found in the values without and with contralateral acoustic stimulation in groups 1 and 2.

**Table 2:** Post hoc comparisons.

Difference	Estimate	Standard error	Df	T	p*	Confidence interval* (95%)	
						Lower limit	Upper limit
G1-G2 SR	1,64	1,09	56,2	1,51	1,000	-1,88	5,17
G1-G3 SR	3,20	1,21	56,7	2,64	0,268	-0,73	7,12
G1-G4 SR	3,31	1,13	58,8	2,93	0,120	-0,34	6,97
G1-G5 SR	4,62	1,14	57,7	4,07	0,003	0,94	8,30
G2-G3 SR	1,55	1,16	56,7	1,34	1,000	-2,19	5,30
G2-G4 SR	1,67	1,07	59,1	1,56	1,000	-1,79	5,13
G2-G5 SR	2,98	1,08	57,8	2,77	0,190	-0,51	6,47
G3-G4 SR	0,12	1,20	59,0	0,10	1,000	-3,75	3,98
G3-G5 SR	1,43	1,20	57,9	1,19	1,000	-2,46	5,32
G4-G5 SR	1,31	1,12	60,5	1,17	1,000	-2,30	4,93
G1-G2 CR	1,83	1,07	55,7	1,70	1,000	-1,65	5,30
G1-G3 CR	2,99	1,19	56,6	2,51	0,373	-0,87	6,85
G1-G4 CR	3,00	1,11	58,6	2,70	0,228	-0,60	6,60
G1-G5 CR	3,98	1,12	57,5	3,56	0,018	0,36	7,61
G2-G3 CR	1,17	1,14	56,7	1,03	1,000	-2,52	4,85
G2-G4 CR	1,18	1,05	59,0	1,12	1,000	-2,23	4,58
G2-G5 CR	2,16	1,06	57,7	2,04	1,000	-1,27	5,59
G3-G4 CR	0,01	1,17	59,4	0,01	1,000	-3,79	3,81
G3-G5 CR	0,99	1,18	58,3	0,84	1,000	-2,83	4,81
G4-G5 CR	0,98	1,10	60,8	0,89	1,000	-2,57	4,54
SR-CR G1	0,68	0,19	45,1	3,55	0,023	0,05	1,31
SR-CR G2	0,86	0,17	45,1	5,03	0,003	0,30	1,42
SR-CR G3	0,47	0,22	46,2	2,16	0,910	-0,25	1,19
SR-CR G4	0,37	0,20	55,4	1,82	1,000	-0,29	1,03
SR-CR G5	0,04	0,20	50,4	0,20	1,000	-0,60	0,68
RE-LE	0,70	0,32	55,1	2,17	0,040	0,05	1,35

Key: Df: degree of freedom; T: t statistics; p: probability

## DISCUSSION

The study revealed a statistically significant difference in the result values of transient evoked otoacoustic emissions without contralateral acoustic stimulation in group 1 and 5 subjects, as shown in Table 2. The value differences in the results of transient evoked otoacoustic emissions without contralateral acoustic stimulation were not statistically significant. Results revealed that outer hair cell activity was more intense in younger subject because there were more cells. As age advances, outer hair cells are lost because of intrinsic or extrinsic factors, which decreases electromotility. In this study, the function of the medial olivocochlear efferent tract in both sexes was not investigated.

The reduction/suppression effect of otoacoustic emissions has been studied in detail,^10^,^11^,^1^,^12^,^7^ and should be present in normal-hearing subjects.^13^ This study ratified this statement, since reduction/suppression of otoacoustic emissions occurred in most subjects.

The reduction/suppression effect of transient evoked otoacoustic emissions was more evident in groups 1 and 2, as shown in Table 2; this reflects the effective function of the physiologically and anatomically intact medial olivocochlear efferent tract in this age group. However, the researchers stated that aging normal-hearing subjects might present changes in the physiological activity of the medial olivocochlear efferent tract.

Studies have suggested that aging causes general changes in anatomical/structural areas - including the auditory system - at an afferent and efferent level.^4^ A study by Gulya has shown that the medial olivocochlear efferent tract, which originates in the superior olivary complex, is affected by the aging process; changes in central auditory structures include the point of origin of this tract.^5^

After age 40 years,4 aging is reflected throughout the auditory system in the production (afferent) and control (efferent) mechanisms of otoacoustic emissions.

Results showed that there was a statistically significant difference among the values of transient evoked otoacoustic emissions without and with contralateral acoustic stimulation in groups 1 and 2, as shown in Table 2. The reduction/suppression effect was present in the sample, but decreases substantially with age. Thus, the otoacoustic emissions control activity of the medial olivocochlear efferent tract is fully operational until around age 40 years, decreasing thereafter with the natural degeneration process of auditory efferent structures, especially the medial olivocochlear efferent tract, in which the axonal myelin sheath becomes partly lost. These results reinforce the hypothesis of a physiological decrease in the effectiveness of the medial olivocochlear efferent tract.^6^,^7^,^4^

Prior studies with similar objectives as this study are scarce; those that have been done arrived at similar results, conclusions and inferences. However, a direct comparison between Castor et al.'s and Parthasarathy's results and those in this study is not feasible, given the different methods.^6^,^7^ These authors inferred that the medial olivocochlear efferent tract may lose function with age. Their results showed that suppression was more evident in younger adults, compared to elderly subjects.

Studies of the efferent auditory pathway raise many questions that remain unanswered, which demonstrates the need for detailed knowledge of this anatomically, physiologically and clinically complex pathway. Further studies may, therefore, investigate particularly the medial olivocochlear efferent tract for additional understanding and clinical application to improve the audiological diagnosis.

## CONCLUSION

The conclusions of this study were:

The reduction/suppression effect of transient evoked otoacoustic emissions was more evident in group 1 and 2 subjects. The results of transient evoked otoacoustic emissions without contralateral acoustic stimulation in groups 1 and 5 showed significant statistical differences; similarly with the results of transient evoked otoacoustic emissions without contralateral acoustic stimulation in group 1 and 2 subjects.

Reduction/suppression of transient evoked otoacoustic emissions decreases substantially with age, starting at about the fourth decade of life; this suggests that the effectiveness of the medial olivocochlear efferent tract acting on the mechanical activity of the external hair cells of the Corti organ is compromised. Aging, thus, affects this activity.
